# Saponins of Marsdenia Tenacissima promotes apoptosis of hepatocellular carcinoma cells through damaging mitochondria then activating cytochrome C/Caspase-9/Caspase-3 pathway

**DOI:** 10.7150/jca.72601

**Published:** 2022-07-04

**Authors:** Xiao-pei Jiang, Shuai Jin, Weiting Shao, Lin Zhu, Shuanggen Yan, Jingtao Lu

**Affiliations:** 1 School of Life Sciences, Anhui Medical University, Hefei, China.; 2 Department of Orthopaedic Surgery, The First Affiliated Hospital of Anhui, Medical University, Hefei, China.

**Keywords:** Saponins of Marsdenia Tenacissima, hepatocellular carcinoma, apoptosis, mitochondria, cytochrome C, Caspase9, Caspase3

## Abstract

Liver cancer is one of the most common cancers in the world and the second leading cause of death in cancer patients. There is an urgent need for an effective and less toxic treatment for liver cancer. Saponins of Marsdenia Tenacissima (SMT) as a potential anticancer drug has attracted extensive attention of researchers because of its effective biological activity. The effect of SMT on HepG2 Li-7 and L-02 cells was detected by CCK8 assay. At the same time, the apoptosis rate was detected by flow cytometer and laser confocal microscope, and the morphological changes of mitochondria were observed under electron microscope. The levels of bax, cytochrome c, caspase-9, caspase-3, cleaved caspase-3 and protein were detected using Western bolt. Finally, BALB/c was subcutaneously injected with H22 cells to form tumors, and SMT was intragastrically injected to detect the size of the transplanted tumor. SMT can induce apoptosis *in vitro* and reduce the size of transplanted tumor *in vivo*. Increases the rate of apoptosis through the cytochrome c pathway and regulates the expression of apoptosis-related proteins. These results suggest that SMT may be one of the potential candidates for the treatment of liver cancer.

## Introduction

Hepatocellular carcinoma (HCC) accounts for the majority of primary liver cancers. Worldwide, Liver cancers are the fourth most common cause of cancer-related deaths, ranking sixth in all incident cases 1. HCC is an aggressive malignancy with few effective treatment options 2. Liver cancer patients are often at an advanced stage of the disease when they are diagnosed. They usually use surgical resection, orthotopic liver transplantation or local percutaneous tumor ablation, but these methods cannot radically remove or ablate the tumor 3, 4, so it is best to use chemotherapy treatment, so its chemotherapy drugs have become a research hotspot 5.

Marsdenia tenacissima (Family Asclepiadaceae) is a perennial climber that is extensive distributed in tropical to subtropical areas in Asia, primarily in the Yunnan and Guizhou Provinces of China 6. MT is a traditional Chinese herbal medicine, which has the effects of dispelling rheumatism, promoting menstruation, promoting blood circulation, and hemostasis. Indications of rheumatic arthralgia, irregular menstruation, bruises, fractures, traumatic bleeding 7, 8. SMT (Saponins of Marsdenia Tenacissima) is an effective part extracted from Marsdenia Tenacissima. Although the anti-tumor effect of SMT in liver cancer has been confirmed, its underlying molecular mechanism is still not fully understood.

The pathway of cell apoptosis is generally divided into two kinds of apoptotic pathways, one is the external pathway that directly activates caspase-8 by cell surface receptors, and the other is the pathway that is internally regulated by mitochondria 9, 10. The apoptosis regulation stage of mitochondria is located upstream of caspase activation, and mitochondrial damage will regulate the release of cytochrome C 11, 12. When cytochrome C is released from the mitochondria, it will bind to APAF-1 to activate the assembly of the apoptotic body of caspase-9, thereby activating the activation of caspase-3 and leading to cell apoptosis 13-15.

## Materials and Methods

### Reagents and Antibodies

Saponins of Marsdenia Tenacissima (>99% pure) was a gift from Institute of Clinical Pharmacology, Anhui Medical University, was dissolved in phosphate buffered saline (PBS) to prepare 10 mg/ml stock solution and stored at -20 °C. PBS was used as vehicle control. Primary antibodies against caspase-9, caspase-3, cytochrome C and cleaved-caspase-3 were purchased from Zen Bio (Chengdu, China), and primary antibodies against Bax were purchased from cohesion biosciences (UK).

### *In vitro* cell culture/maintenance

The Li-7 and HepG2 human HCC cell lines, the L-02 human liver cell line, and H22 murine HCC cell line were purchased from purchased from Shanghai Cell Bank, Chinese Academy of Science (Shanghai, China). HepG2 human HCC cell line was cultured in DMEM Medium with 10% fetal bovine serum (VivaCell, Shanghai, China), 100 U/mL penicillin, and 100 μg/mL streptomycin and Li-7 human HCC cell line, L-02 human liver cell line and H22 murine HCC cell line were cultured in RPMI 1640 Medium with 10% fetal bovine serum, 100 U/mL penicillin, and 100 μg/mL streptomycin in a humidified atmosphere containing 5% CO_2_ at 37 °C.

### Animals

Male BALB/c mice (body weight: 18-22 g) were provided by the Experimental Animal Center of Anhui Medical University (Hefei, China). Animals were maintained in a pathogen-free environment (23±2 °C, 55±5% humidity) on a 12-h light/dark cycle throughout the experimental period. All animal studies were approved by the Institutional Animal Care and Use Committee of Anhui Medical University.

### Cell treatment

Li-7 cells, HepG2 cells and L-02 cells were seeded in a six-well plate at a density of 2×10^6^ cells/mL. Subsequently, SMT (125, 250, and 500 μg/mL) was added, and the cells were incubated for 24 hours. The control group was added with the same medium without SMT. After SMT treatment, Li-7 cells, HepG2 and L-02 were harvested and used in the following experiments.

### Cell Counting Kit-8 (CCK-8) assay

The *in vitro* inhibitory effects of M. tenacissima were determined by CCK-8 assay. Briefly, exponentially growing Li-7, HepG2 tumor cells and L-02 liver cells were seeded into a 96-well microtiter plate at a density of 5 × 10^3^ cells/well, and then stabilized overnight, then different concentrations of SMT (62.5, 125, 250, 500, 1000 μg/ml) incubate for 24 hours and 48 hours.Before measurement, cells were incubated with CCK-8 at 37 °C for 1 h. Finally, the absorbance of each well was determined at 450 nm.

### CLSM

Li-7 cells were cultured in RPMI-1640 medium with 10% FBS, and HepG2 cells were cultured in DMEM medium with 10% FBS. The cells were treated with SMT (125, 250, 500 μg/ml) for 24 hours, and the apoptosis of Li-7 cells and HepG2 cells was analyzed by CLSM.

### Flow cytometry was used to analyse apoptosis

Li-7 cells were cultured in RPMI-1640 medium with 10% FBS, and HepG2 cells were cultured in DMEM medium with 10% FBS. The cells were treated with SMT (125, 250, 500 μg/ml) for 24 hours, and the apoptosis of Li-7 cells and HepG2 cells was analyzed by flow cytometry.

### Electron microscope observation

After Li-7 cells and HepG2 cells were treated with SMT (250 μg/ml) for 24 hours, the cells were collected, washed twice with phosphate buffered saline (PBS), and transferred to a 1.5 mL EP tube. The cells were washed 3 times with PBS (15min/time), and then fixed with 1% osmotic acid (60 min) and 2% uranyl acetate (30 min). After the cells were dehydrated by a series of gradient ethanol, they were infiltrated with pure acetone and the embedding medium (1:1) for 60 minutes, and then the embedding medium alone was infiltrated for 60 minutes. The samples were dried at 37 °C for 24 hours, 45 °C for 24 hours, and 60 °C for 48 hours to prepare ultrathin sections (0.1 μM) and observe under a transmission electron microscope. 10 cells were randomly selected from each group for mitochondrial observation.

### Western blot analysis

Li-7 cells and HepG2 cells were harvested and lysed in lysis buffer. The protein concentration was measured using the bicinchoninic acid protein determination kit (Vazyme, Nanjing, China), and then heated under reducing conditions. A total of 20 µg of protein was loaded onto a sodium lauryl sulfate-polyacrylamide gel electrophoresis gel and then transferred to a polyvinylidene fluoride membrane. After blocking, the membrane was incubated with the following antibodies: β-catin (1:2,000), cytochrome C (1:1,000), caspase-3 (1:1,000), caspase-9 (1:1,000), bax (1:2,000), cleaved-caspase-3 (1:1,000).

### *In vivo* injection of H22 cells

The exponentially growing H22 cells were injected subcutaneously into the right armpit of BALB/c mice (2.5×10^6^ cells/mouse). Each group includes six animals. On the 3rd day, the mice were intragastrically injected with SMT (60 mg/kg, 120 mg/kg) or normal saline every day. On the 14^th^ day, the mice were sacrificed humanely, and the tumors were taken out and weighed.

### Statistical analysis

The data were expressed as the mean ± standard deviation from at least three independent experiments. Statistical analysis was performed using the statistics software GraphPad Prism 10 (GraphPad Software, Inc., La Jolla, CA). Comparisons between two and multiple groups were performed using Student's *t*-test and one-way analysis of variance, respectively. *P<*0.05 denoted statistically significant differences.

## Results

### Saponins of Marsdenia Tenacissima inhibits the growth of HCC cells

**Figure 1:**
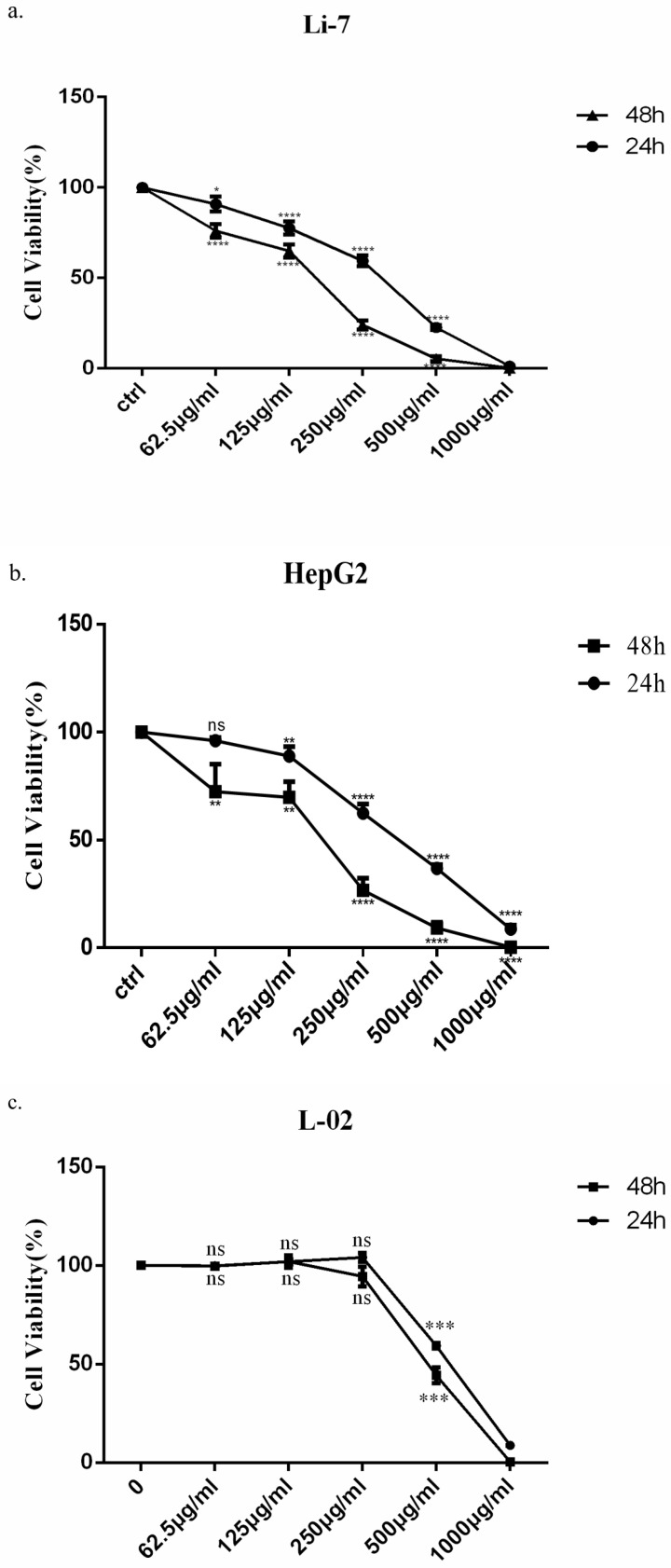
** Saponins of Marsdenia Tenacissima inhibits the growth of HCC cells.** The effect of SMT on the viability of HepG2 and Li-7 cells. **a-c** Cell viability was measured by CCK-8 assay at 24 h and 48 h after SMT treatment in HepG2 Li-7, and L-02 cells. All data are expressed as means ± standard deviation. *P<0.05, **P<0.01, ***P<0.001, ****P<0.0001 *vs.* control group.

In order to observe the effect of SMT on cell growth, HepG2, Li-7 and L-02 cells were incubated with increasing concentrations of SMT, and the number of cells was counted after 24h and 48h. HepG2 cells were more sensitive to SMT than Li-7 cells. However, the killing effect of SMT on the normal liver cell line L-02 was less than that of the HCC cell lines Li-7 and HepG2. The viability of HepG2 and Li-7 cells gradually decreased with the increase of SMT concentration (the IC50 value was calculated as 408.3 μg/mL for Li-7 cells, 290.6 μg/mL for HepG2 cells and 534.1 μg/mL for L-02, 48 h; Fig. 1a,b,c).

### SMT promotes apoptosis of HCC cells

**Figure 2:**
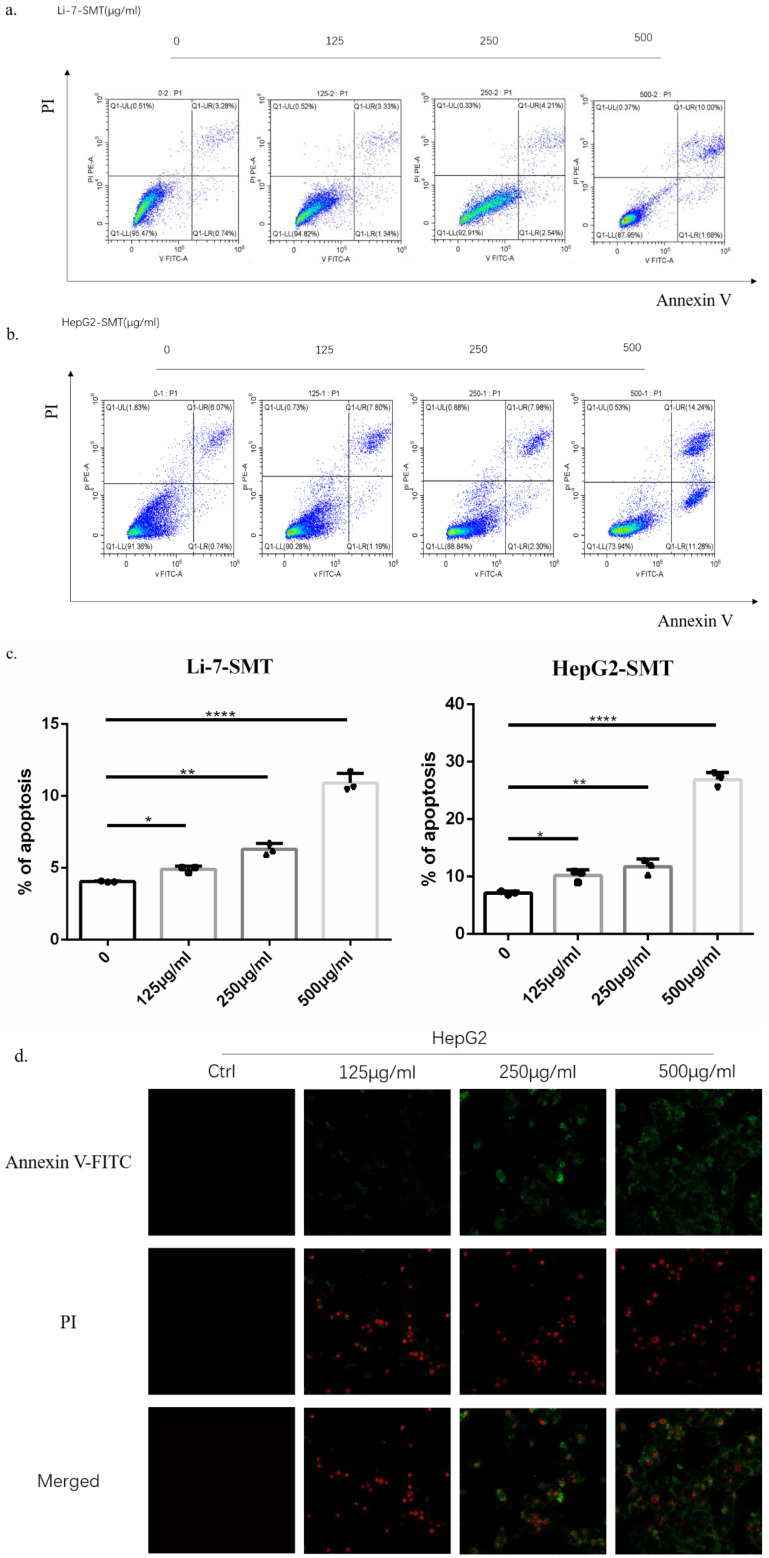

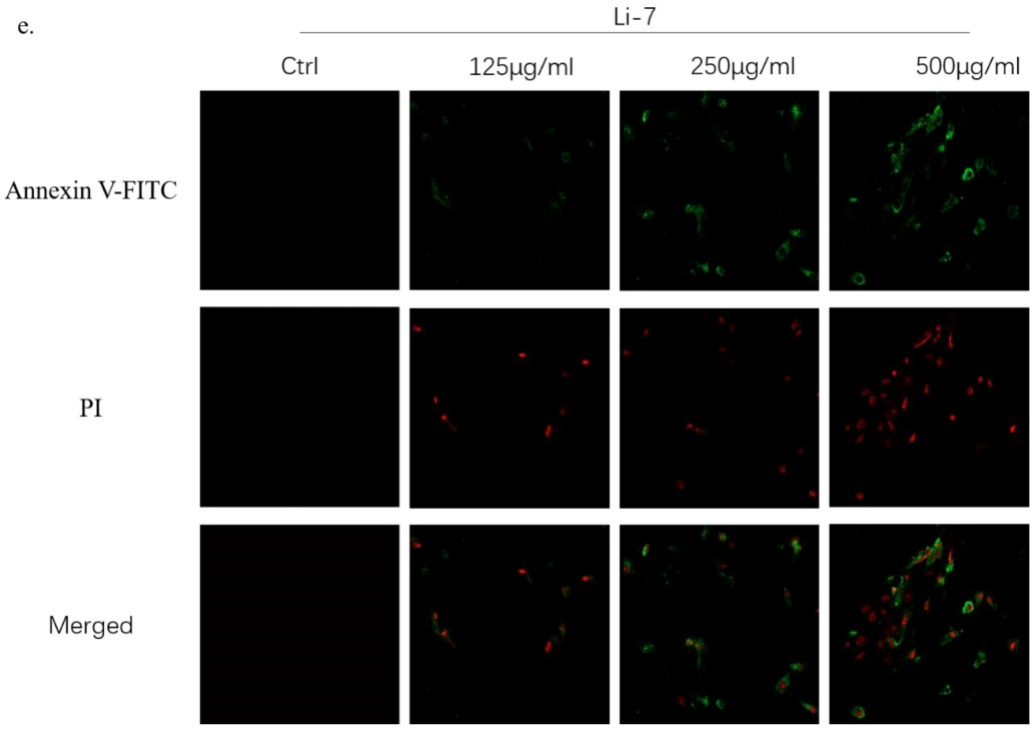
** SMT promotes apoptosis of HCC cells.** Effect of SMT on apoptosis in HepG2 and Li-7 cells. **a-c**.The percentage of apoptosis of Hep-G2 and Li-7402 cells after SMT treatment tested by FLS. All data are expressed as means ± standard deviation. *P<0.05, **P<0.01, ***P<0.001, ****P<0.0001 vs. control group. **d-e**. Confocal laser microscope images of apoptotic cells after Annexin V-FITC/PI double staining. Apoptotic cells are shown in green, while necrotic cells are shown in red (original magnification: ×100).

In order to explore the effect of SMT on the apoptosis of HCC cells, different concentrations of SMT (125, 250, 500 μg/ml) were used to treat liver cancer cells Li-7 cells and HepG2 cells for 24 h. Flow cytometry was used to analyze the apoptosis of liver cancer cells. Apoptosis, the results showed that compared with the untreated group, the apoptosis rate of HepG2 cells and Li-7 cells was significantly increased after 24h of SMT treatment (Fig. 2a,b,c). The apoptosis of hepatoma cells was observed by confocal microscopy. The results showed that compared with the untreated group, the apoptosis rate of HepG2 cells and Li-7 cells was significantly increased after 24 h of SMT treatment (Fig. 2d,e).

### SMT damages the function of mitochondria of HCC cells

**Figure 3:**
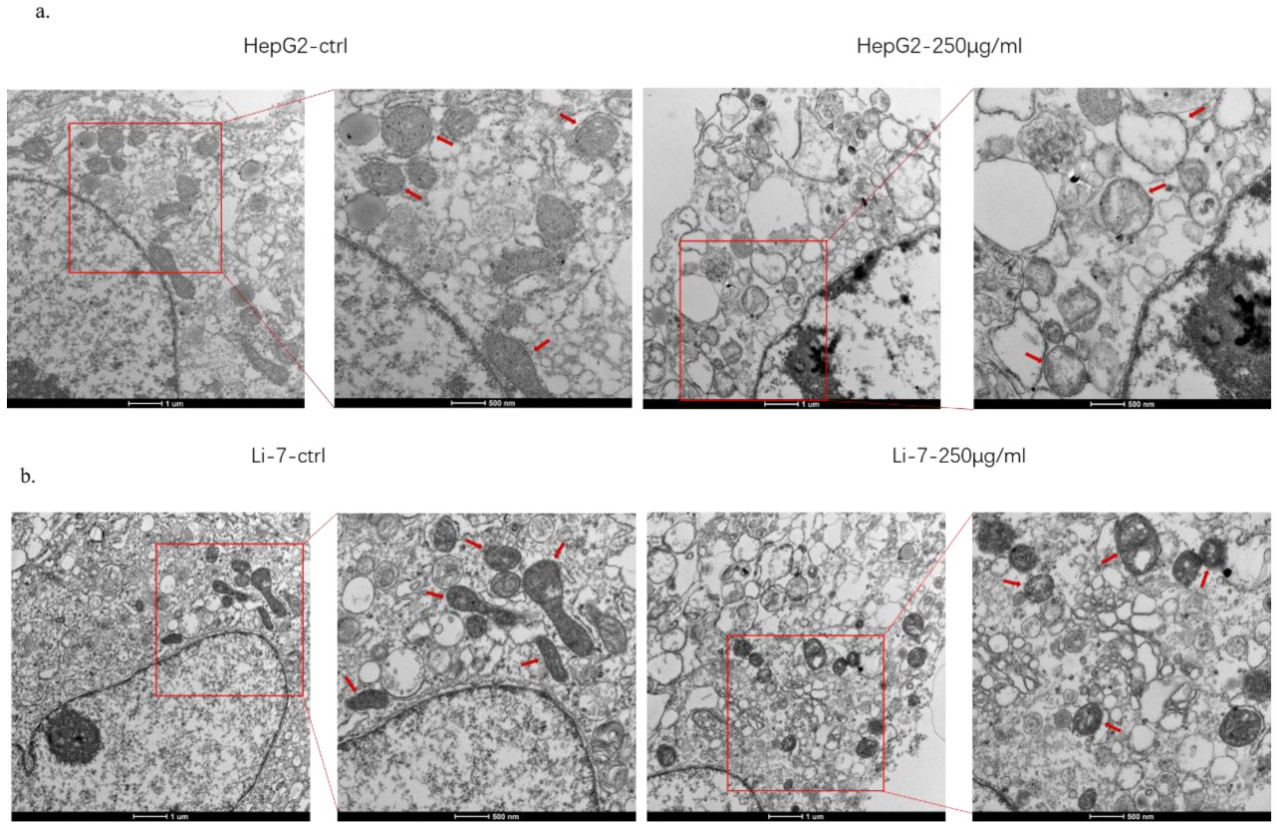
** SMT damages the function of mitochondria of HCC cells.** Effects of SMT on related proteins. **a-b**. Cell viability was measured by transmission electron microscope (TEM) at 24 h after SMT (250 µg/ml) treatment in HepG2 and Li-7 cell.

In order to explore the mechanism of SMT-stimulated apoptosis of hepatocellular carcinoma cells, this study used electron microscopy to observe the structural changes of mitochondria in hepatoma cells. The results showed that when HCC cells were treated with SMT (250 μg/mL) for 24 h, apoptotic HCC cells exhibited mitochondrial swelling and cristae fragmentation, which are striking features of damaged mitochondria (Fig. 3a,b). These results suggest that SMT can impair mitochondrial function in hepatoma cells.

### SMT activates cytochrome C/Caspase9/Caspase3 pathway

**Figure 4:**
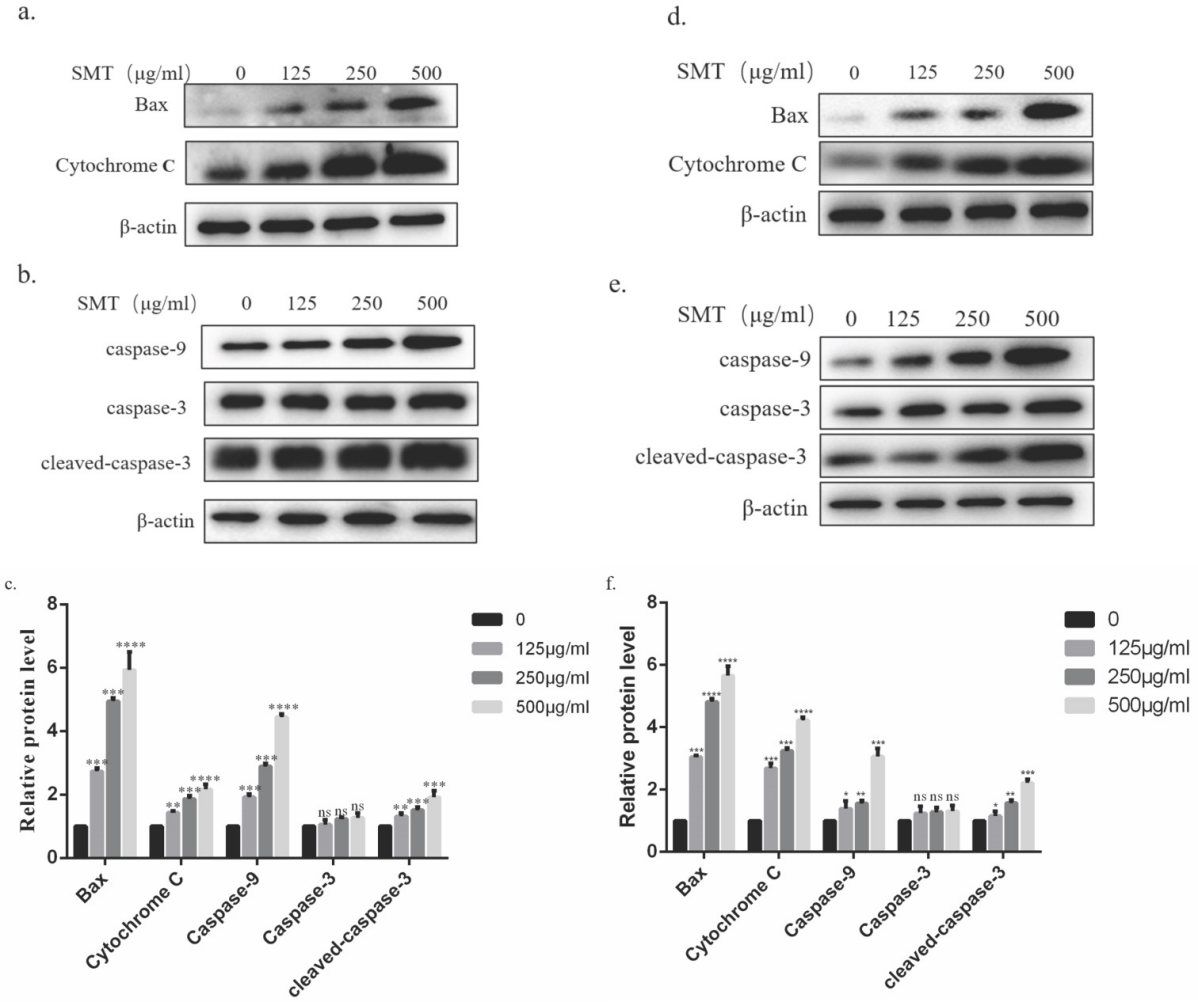
**SMT activates cytochrome C/Caspase9/Caspase3 pathway.** Expression of related proteins. **a-b** Effects of SMT on Bax and Cytochrome C , Caspasse-9, Caspasse-3 and Cleaved Caspase-3, a pathway in HepG2 cells.**c** The histograms show apoptosis-related protein expression in HepG2 cells. **d-e** Effects of SMT on Bax and Cytochrome C, Caspasse-9, Caspasse-3 and Cleaved Caspase-3, a pathway in Li-7 cells. **f** The histograms show apoptosis-related protein expression in Li-7 cells. These results were obtained from three independentexperiments, and all of the data are expressed as the mean ± SD, *p < 0.05, **p < 0.01, ***p < 0.001,****P<0.0001 vs. the control group (n=3).

The possible mechanism of SMT-induced apoptosis was analyzed by Western blotting. The results showed that the expression of BAX, cytochrome C, and caspase-9 in HepG2 and Li-7 cells increased significantly with the increase of SMT concentration. Although the expression level of caspase-3 did not change significantly, the expression level of cleaved-caspase-3 increased significantly (Fig. 4a-d).

### SMT inhibits HCC growth *in vivo*

**Figure 5:**
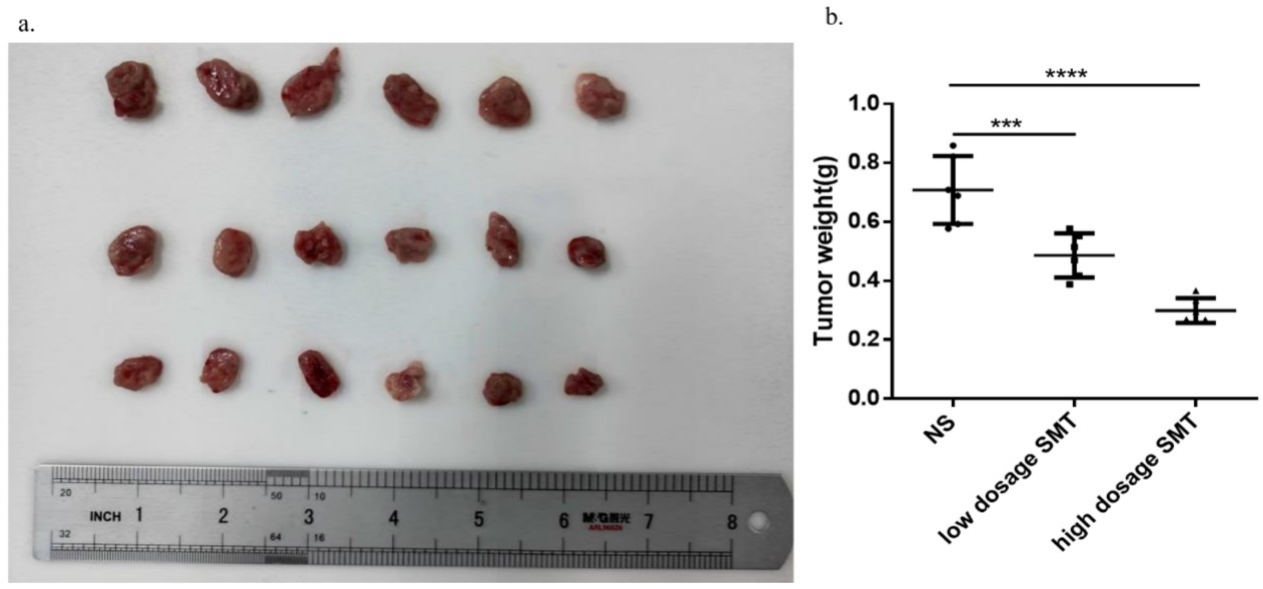
**SMT inhibits HCC growth *in vivo*.** Effects of SMT on HCC cells *in vivo***. a-b**.Subcutaneous tumors extracted from BALB/c mice 14 days after implantation, and the weight of these tumors. n = 3, *P < 0.05, **P < 0.01, ***P < 0.001 vs. control group.

To determine the effect of SMT on HCC development *in vivo*, we subcutaneously transplanted mouse-derived HCC cell line H22 cells into BALB/c mice and injected SMT (60 mg/kg, 120 mg/kg) or saline by gavage 3 days after inoculation. The mice were sacrificed 14 days after transplantation. The results showed that the tumors in the saline-injected mice grew faster than in the SMT-injected mice (Fig. 5a,b).

## Discussion

Liver cancer is the sixth most common malignancy in the world, and most patients are diagnosed in the middle and late stages 2. At present, chemotherapy is the main treatment method for liver cancer, especially advanced patients, but it has a great impact on the immune function of the body 16. In recent years, traditional Chinese medicine has obvious advantages in clinical cancer treatment, such as good curative effect and less side effects. The anticancer drug Xiaoaiping, which uses Marsdeniae tenacissimae as raw material, has been used in the treatment of liver cancer and other tumors, and has achieved good clinical effects 17, 18. However, Xiaoaiping is a crude extract of Marsdeniae tenacissimae, and the clinical dose is large, which is not conducive to patients taking it. In this study, the effect of SMT, an extract of Radix vulgaris, on liver cancer cells *in vitro* and *in vivo* was investigated.

It is well known that the morphological changes and functional damage of mitochondria are important causes of cell death or apoptosis. 11. Mitochondria provide energy for cell growth by promoting metabolism, but their swelling and ridge breakage suggest a loss of mitochondrial function 19, 20. In this study, electron microscope observation showed that mitochondrial swelling and ridge rupture occurred after SMT was applied to human hepatoma cell lines HepG2 and Li-7. These results suggest that SMT can promote hepatoma cell apoptosis, and the mechanism may be related to damage to mitochondrial structure and inhibition of mitochondrial function.

We explored the mechanism by which SMT promotes apoptosis in hepatoma cells. First, using our Annexin V-FITC and propidium iodide (PI) staining, the apoptosis rate was detected by flow cytometry and laser confocal microscopy. The results showed that the apoptosis rate of hepatoma cells increased gradually with the increase of SMT concentration. Subsequently, under the electron microscope, we found that SMT can induce changes in the mitochondrial structure of liver cancer cells. Western blot detected that with the increase of SMT concentration, the expression of Bax in bcl family proteins increased, and the expression of cytochrome c in the cytoplasm led to caspase-9 changes in expression. Interestingly, the total expression of caspase-3 did not change, but the activated caspase-3 increased with increasing concentration. This is consistent with our *in vitro* and *in vivo* experimental results. So we speculated that SMT increased the expression of its cleaved caspase-3, leading to apoptosis of HCC cells.

**Figure 6:**
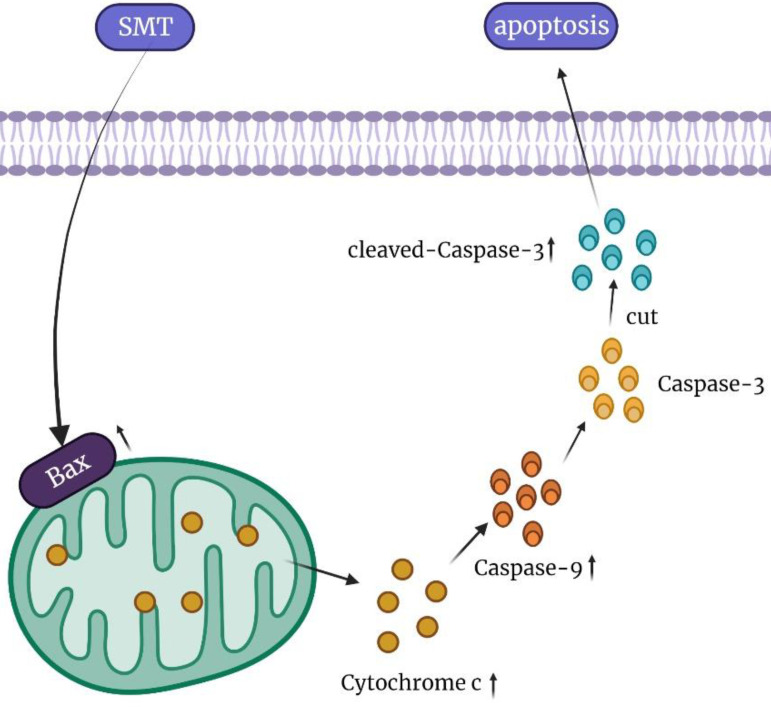
Summary of the mechanism of SMT promoting apoptosis of hepatocellular carcinoma cells.

In conclusion, our results show that SMT at a certain concentration can promote the apoptosis of HCC cells, but not normal hepatocytes. The mechanisms involved in this process may be related to mitochondrial damage and increased cytochrome c expression (Figure 6). In the present study, SMT-induced apoptosis of hepatoma cells was an important molecular mechanism for inhibiting proliferation *in vitro* and *in vivo*. In addition to down-regulating the caspase-3 apoptotic pathway, we speculate that there are other apoptotic mechanisms that require further study in the future. This study provides a new experimental basis for the application of SMT in the clinical treatment of liver cancer.
